# Preventable hospitalizations through ED: does the number of hospital beds matter under the global budget in a single-payer system in Taiwan?

**DOI:** 10.3389/fpubh.2024.1460270

**Published:** 2025-01-06

**Authors:** Hsueh-Fen Chen, Hui-Min Hsieh, Wei-Shan Chang

**Affiliations:** ^1^Department of Healthcare Administration and Medical Informatics, College of Health Sciences, Kaohsiung Medical University, Kaohsiung City, Taiwan; ^2^Department of Medical Research, Kaohsiung Medical University Hospital, Kaohsiung Medical University, Kaohsiung City, Taiwan; ^3^Center for Big Data Research, Kaohsiung Medical University, Kaohsiung City, Taiwan; ^4^Department of Public Health, College of Health Sciences, Kaohsiung Medical University, Kaohsiung City, Taiwan; ^5^Division of Medical Statistics and Bioinformatics, Department of Medical Research, Kaohsiung Medical University Hospital, Kaohsiung Medical University, Kaohsiung City, Taiwan

**Keywords:** ambulatory care sensitive conditions, treat-and-leave emergency department visits, global budget, preventable hospitalizations, diabetes-related complications, floating-point value

## Abstract

**Background:**

Taiwan implemented global hospital budgeting with a floating-point value, which created a prisoner's dilemma. As a result, hospitals increased service volume, which caused the floating-point value to drop to less than one New Taiwan Dollar (NTD). The recent increase in the number of hospital beds and the call to enhance the floating-point value to one NTD raise concerns about the potential for increased financial burden without adding value to patient care if hospitals expand their bed capacity for volume-based competition. The present study aimed to examine the relationship between the supply of hospital beds and hospitalizations following an emergency department (ED) visit (called ED hospitalizations) by using diabetes-related ambulatory care sensitive conditions (ACSCs) that are preventable and discretionary as an example.

**Methods:**

The study was a pooled cross-sectional design analyzing 2011–2015 population-based claims data in Taiwan. The dependent variable was a dummy variable representing an ED hospitalization, with a treat-and-leave ED visit as the reference group. The key independent variable is the number of hospital beds per 1,000 populations. Multivariate logistic regression models with and without a clustering function were used for the analyses.

**Results:**

Approximately 59.26% of diabetes-related ACSCs ED visits resulted in ED hospitalizations. The relationship between the supply of hospital beds and ED hospitalizations was statistically significant (OR = 1.12; 95% CI: 1.09–1.14; *P* < 0.001) in the model without clustering but was statistically insignificant in the model with clustering (OR = 1.03; 95% CI: 0.94–1.12; *P* > 0.05). Several social risk factors were positively associated with the likelihood of ED hospitalizations, such as low income and the percentage of the population without a high school diploma. In contrast, other factors, such as female patients and the Charlson comorbidity index, were negatively associated with the likelihood of ED hospitalizations.

**Conclusion:**

Under hospital global budgeting with a floating-point value mechanism, increases in hospital beds likely motivate hospitals to admit ED patients with preventable and discretionary conditions. Our study emphasizes the urgent need to add value-based incentive mechanisms to the current global budget payment. The value-based incentive mechanisms may encourage providers to focus on quality of patient care by addressing social risk factors rather than engage in volume-based competition, which would improve population health while reducing preventable ED visits and hospitalizations.

## Introduction

Hospital expenditure is the largest share of total healthcare spending in many countries (1, 2). Payers have used hospital global budgeting to contain escalating expenditures by sharing financial accountability with hospitals and encouraging hospitals to work with other providers to reduce unnecessary care or preventable hospitalizations (3–6). There are different forms of global budgeting. For example, the Maryland Total Cost of Care Model in the United States implemented a global budget without price adjustments for each service, while Taiwan and Germany implemented a global budget with price adjustments (7). The variations in global budgeting would likely affect providers' behaviors differently.

In 1995, Taiwan implemented the national health insurance (NHI) program with a single-payer system, operated by the National Health Insurance Administration (NHIA). At the beginning of NHI, providers were paid by the fee-for-service payment system. Because of the fast-growing health expenditure, the NHIA adopted the global budget payment with price adjustments through a floating-point value mechanism and gradually applied the payment to dental, Chinese medicine, and Western medicine in clinics and hospitals in 1998, 2000, 2001, and 2002, respectively. The amount of the global budget nationally depends on the total expenditure in the previous year, the change in population characteristics and providers' operating costs, the growth of the economy, and policies, such as care for high-risk populations and remote areas (8).

The features of the global budget payment with a floating-point value mechanism in Taiwan are a mixed prospective and retrospective payment. The amount of budget to pay providers is predetermined before the start of the calendar year, which is prospective. A floating-point value is equivalent to the total predetermined budget divided by the total service volume rendered by all providers in the market, which is retrospective. Using the hospital industry as an example, an individual hospital's total payment is a floating-point value multiplied by the total service volume delivered by the hospital. Given a floating-point value mechanism, hospitals do not know their total payment until the NHIA counts the service volume from all hospitals and calculates a floating-point value per service volume.

Hospital global budgeting with a floating-point value mechanism creates the phenomenon called prisoner's dilemma to hospitals (9, 10), because the total payment to an individual hospital depends on the volume of services provided by the hospital and other hospitals in the same market. Ideally, it would be good if all hospitals agreed to produce a specific volume to keep a floating-point value at their desired value. However, an individual hospital is unlikely to know the other hospitals' service volume; therefore, an individual hospital is willing to increase its service volume to secure its share from the predetermined budget. Empirical evidence from Taiwan showed that, in response to the challenges of prisoner's dilemma, hospitals engaged in volume-based competition. This included increasing services with high price/cost margins (e.g., radiology), as well as boosting the number of prescriptions, procedures, and length of stays (9–11). Due to volume-based competition, the floating-point value was, on average, less than one New Taiwan Dollar (NTD).

Hospitals may also increase their capacities, allowing them to compete with others regarding service volume. Recently, medical centers and chain hospitals in Taiwan have expanded the number of hospital beds. More than 3,000 new hospital beds are either under reconstruction or have been approved and will soon enter the market, reaching 4.5 hospital beds per 1,000 populations. This would be higher than the average of 4.3 hospital beds per 1,000 populations in the Organization for Economic Cooperation and Development (OECD) countries (12). However, evidence regarding the association between the supply of hospital beds and hospitalization rates, especially for emergency department (ED) hospitalizations under the global budget with the price adjustment payment system, is limited.

The present study aimed to examine the association between the supply of hospital beds and ED hospitalizations. We chose diabetes-related ambulatory care sensitive conditions (ACSCs) as an example because ED hospitalizations due to diabetes-related ACSCs are preventable and discretionary. Defined by the Prevention Quality Indicators (PQI) program in the Agency for Healthcare Research and Quality (AHRQ), diabetes-related ACSCs include short-term complications (e.g., ketoacidosis with/without coma), long-term complications (e.g., diabetic nephropathy or retinopathy), uncontrollable diabetes (hypo/hyper-glycemia with or without coma), and lower-extremity amputation. Hospitalizations due to those conditions are theoretically preventable if patients can receive proper care in the community. Furthermore, there is a high variation in ED physicians' decisions regarding ED admissions, which cannot be explained by patients' clinic conditions (13–15). Thus, ED hospitalizations due to diabetes-related ACSCs are considered discretionary.

### Significance of the study

The concept of “supply induces demand” is commonly supported by studies based on the fee-for-service payment system, activity-based payment, or diagnosis-related group (DRG) payment (16–22). However, the results of ED hospitalizations are mixed. For example, in England, O'Cathain et al. (21) examined a broad definition of preventable hospitalizations (e.g., cellulitis and injury) at the hospital level and found that the supply of hospital beds was positively associated with the likelihood of ED hospitalizations. By using all ED conditions, the study from Sweden showed that ED patients were likely to be hospitalized when hospital beds were available (14); however, the study from the Medicare fee-for-service population in the United States showed a negative association between the supply of hospital beds and ED hospitalizations (22, 23). As many countries have moved payment systems away from the fee-for-service payment toward a global budget payment (14, 24), testing a theoretical assumption about the prisoner's dilemma by utilizing preventable and discretionary conditions is needed.

With the challenge of unpredictable revenue under the predetermined budget payment with price adjustments through a floating-point value mechanism, hospitals in Taiwan provide a good case to test the theoretical assumption (9, 10). Based on previous evidence in Taiwan (9–11, 25), we hypothesized a positive association between the supply of hospital beds and the likelihood of ED hospitalizations for preventable and discretionary conditions. Most importantly, the NHIA in Taiwan faces a significant challenge from providers that call for enhancing the budget to pay hospitals by increasing a floating-point value from less than 1 NTD to 1 NTD. If the supply of hospital beds is positively associated with discretionary and preventable hospitalizations, increasing the floating-point value would increase the financial burden on the NHIA without adding the value of care for patients. The findings in the present study could have important implications for healthcare policies.

## Materials and methods

### Data sources

The present study used six data sources: (1) the 2011–2015 National Health Insurance Research Database (NHIRD), (2) the Medical Care Institution file, (3) the Ministry of Interior Global Information Network (GIN), (4) the Taiwan Medical Association, and (5) DATA.GOV.TW, a platform that comprises various data at the township and district levels. The NHIRD provided enrollment and inpatient and outpatient claims data at the patient level. The enrollment file provides the insured's sociodemographic characteristics and residential locations. The inpatient and outpatient claims data that provide the International Classification of Disease 9th Revision, Clinical Modification (ICD-9-CM) for up to three diagnoses and procedures were used to identify ED hospitalizations and patients who were discharged from ED (hereafter called treat-and-leave ED visits) due to diabetes-related ACSCs, respectively. The Medical Care Institution File provides information on whether patients received care at medical centers, regional hospitals, or local hospitals. The GIN provides data concerning the number of individuals with different levels of education and the number of Aboriginginal people in each township. The Taiwan Medical Association provides the number of physicians in the workforce. Finally, DATA.GOV.TW provides the number of hospital beds at the township/district level.

### Study design and study sample

**Figure 1:**
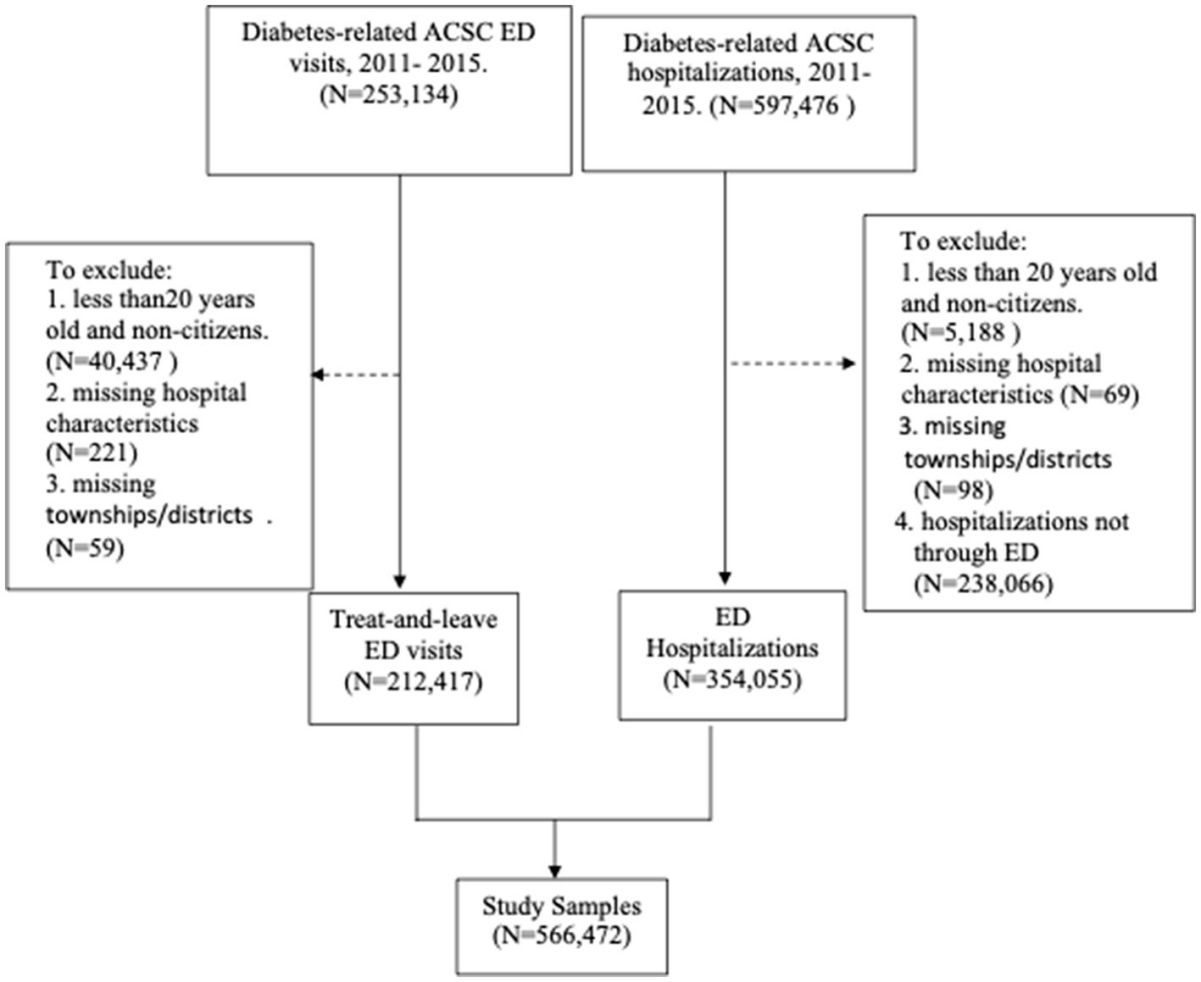
Flow chart of the selection process for the study sample.

The study design was a pooled cross-sectional study using data from 2011 to 2015. We selected this period because the NHIRD started providing the code of ED hospitalizations in the “Patient_source” starting in 2011, and diagnosis codes were coded by ICD-9-CM prior to 2016. The study sample was ED hospitalizations (extracted from inpatient files) and treat-and-leave ED visits (extracted from outpatient files) for individuals aged 20 and above with diabetes-related ACSCs. We applied the ICD-9-CM codes listed by the AHRQ PQI program to identify hospitalizations and ED visits due to diabetes-related ACSCs. We excluded observations with missing data in residential locations and institutions. For the ED hospitalization group, the observations without the code “Patient_source” as ED were excluded. We then merged the ED hospitalizations and treat-and-leave ED visits to form the study sample in the present study. The selection process of the study sample is shown in Figure 1.

### Variable measures

#### Dependent variables

##### ED hospitalizations due to diabetes-related ACSCs

Figure 1 shows two groups of patients—ED hospitalizations and treat-and-leave ED visits—in the study sample. We created a dichotomous variable for observations with ED hospitalizations, with observations in the treat-and-leave ED visits group as the reference group.

#### Key independent variable

The key independent variable is the supply of hospital beds, which is measured as a continuous variable by the number of general hospital beds per 1,000 populations at the township/district level.

##### Control variables

Based on the ACSC-related literature, the likelihood of ED hospitalizations is associated with patients' health conditions and the context of organizations and communities (13, 14, 22, 26–28). Thus, these variables were included in the analytical models. Patient characteristics included age (50–64, 65–79, and 80+, with 20–49 as the reference group), sex (female, with male as the reference group), ethnicity, low income, education level, and the Charlson comorbidity index (29). Ethnicity was proxied by a dummy variable representing the townships/districts with the percentage of Aboriginal people at the 75 percentile and above. A dummy variable measured low-income status if individuals qualified for free premiums. Education was proxied by the percentage of the population without a high school diploma at the township/district level. Organization factors included two dummy variables: regional and local hospitals, with medical centers as the reference group. Community characteristics included the number of primary care physicians per 1,000 populations and two dummy variables representing rural and suburban areas, with urban as the reference group based on the findings from Liu et al.'s (30) study. Finally, we also added year dummies, with 2010 as the reference group to capture potential changes due to time.

### Analytical approach

We conducted *t*-tests for continuous variables and chi-squared tests for categorical variables to compare the differences in study variables between ED hospitalizations and treat-and-leave ED visits. To observe the likelihood of ED hospitalizations associated with the supply of hospital beds, we applied multivariate logistic regression models with ED visits as the unit of analysis. We gradually added the characteristics of patients, hospitals, and communities. Theoretically, the likelihood of ED hospitalizations is affected by hospital policies, the ED's crowdedness, and the community's context (e.g., resources and support systems), which cannot be completely measured in the current study (13, 15, 23, 31). To consider these contextual factors, we conducted sensitivity analyses with clustering patients at the hospital and community levels. A *P*-value of < 0.05 is considered significant. Statistical analyses were performed using SAS v 9.4 (SAS Institute, Cary, NC).

## Results

In the 2011–2015 outpatient files, we identified 253,134 ED visits due to diabetes-related ACSCs. After excluding patients aged under 20 years or non-citizens (*N* = 40,437), those with missing township/district data (*N* = 59), and those with missing hospital information for ED visits (*N* = 221), the total number of ED visits included in the analytical model was 212,417. In the 2011–2015 inpatient files, we identified 597,476 diabetes-related ACSCs admissions. We excluded patients aged under 20 years or non-citizens (*N* = 5,188), hospitalizations not through ED (*N* = 238,066), patients with missing township/district data (*N* = 98), and missing hospital information (*N* = 69). The total number of ED hospitalizations was 354,055. After merging the qualified sample from the inpatient and outpatient files, the final study sample used in the study was 566,472. Figure 1 presents the selection process of the study sample.

Table 1 presents the descriptive statistics of the study variables between ED hospitalizations and treat-and-leave ED visits. The ED hospitalization rate was ~59.26% (354,055/597,476 in Figure 1). The differences in all the study variables between treat-and-leave ED visits and ED hospitalizations were significant (*P* < 0.05). The number of hospital beds per 1,000 populations in the treat-and-leave ED group was 0.45, while in the ED admission group, it was 0.59. However, the differences in certain variables (e.g., low income or Aboriginal status) between the two groups were small but significant, which is likely due to the large number of observations in the present study. For example, the ED hospitalization group included 3.96% low-income patients and 19.67% Aboriginal individuals, while the treat-and-leave ED visit group had 3.47% low-income patients and 20.91 % Aboriginal individuals (*P* < 0.001). Surprisingly, the average Charlson comorbidity index was slightly higher in the treat-and-leave ED group (4.48) than in the ED hospitalization group (4.05). Regional hospitals had ~48% of the treat-and-leave ED visit group and 52% of the ED hospitalization group.

**Table 1:** Descriptive statistics between treat-and-leave ED visits and hospitalizations through ED due to diabetes-related ACSCs (percentage in the parenthesis).

**Study variables**	**Treat-and-leave ED (*n* = 214,417)**	**Hospitalizations through ED (*n* = 354,055)**	***P*-value**
**Key independent variable**			
Hospital beds per 1,000 populations^a^	0.45 ± 0.60	0.59 ± 0.09	< 0.001^***^
**Years**			
Year 2011	43,236 (20.35%)	75,697 (21.38%)	< 0.001^***^
Year 2012	42,715 (20.11%)	72,942 (20.60%)	
Year 2013	41,312 (19.45%)	68,221 (19.27%)	
Year 2014	42,551 (20.03%)	68,080 (19.23%)	
Year 2015	42,603 (20.06%)	69,115 (19.52%)	
**Individual factors**			
Aged 20–49	29,636 (13.95%)	42,578 (12.03%)	< 0.001^***^
Aged 50–64	59,994 (28.24%)	96,529 (27.26%)	
Aged 65–79	75,810 (35.69%)	129,302 (36.52%)	
Aged 80+	46,977 (22.12%)	85,646 (24.19%)	
Female	104,005 (48.96%)	172,040 (48.59%)	0.007^**^
Male	108,412 (51.04%)	182,015 (51.41%)	
Township with high number of Aboriginal people (≥75th P)	44,427 (20.91%)	69,637 (19.67%)	< 0.001^***^
Township with low number of Aboriginal people (< 75th P)	167,990 (79.09%)	284,418 (80.33%)	
Low income	7,371 (3.47%)	14,022 (3.96%)	
Percentage of population without a high-school diploma^a^	0.30 ± 0.10	0.31 ± 0.10	
Charlson comorbidity index^a^	4.48 ± 2.49	4.05 ± 2.59	
**Hospital characteristics**			
Regional hospital	102,097 (48.06%)	18,5334 (52.35%)	< 0.001^***^
District hospital	47,910 (22.55%)	77,164 (21.79%)	
Medical center	62,410 (29.38%)	91,557 (25.86%)	
**Community characteristics**			
Primary care physicians per 1,000 populations^a^	0.08 ± 0.10	0.08 ± 0.09	< 0.001^***^
Rural	92,786 (43.68%)	144,596 (40.84%)	
Suburban	92,276 (43.44%)	155,440 (43.90%)	
Urban	27,355 (12.88%)	54,019 (15.26%)	

^a^Mean and standard deviation in the parenthesis.

^*^P < 0.05.

^**^P < 0.01.

^***^P < 0.001.

Table 2 shows the results of four models without clustering. Model 1 includes the number of hospital beds per 1,000 populations and dummy variables for the years 2012–2015 only. Models 2 to 4 are based on Model 1 by gradually adding the characteristics of patients, hospitals, and communities. The odds ratio of the number of hospital beds per 1,000 populations in Model 1 was less than one and significant (OR = 0.94, 95% CI: 0.94–0.95; *P* < 0.001). However, in Models 2 and 3, the odds ratios were greater than one and insignificant (OR = 1.01 for both models, 95% CI: 0.99–1.02 in Model 2, and 95% CI: 1.00–1.02 in Model 3; *P* > 0.05). In Model 4 that is the full model, the odds ratio of the number of hospital beds per 1,000 populations was significant (OR = 1.12, 95% CI: 1.09–1.14; *P* < 0.001), indicating that after controlling for covariates, increases in one hospital bed per 1,000 populations are associated with increases in 12% likelihood of ED hospitalizations. Table 3 shows the results of four models with clustering at the hospital and community levels. The odds ratio of the number of hospital beds per 1,000 populations in Models 1–4 was similar to that in Table 2, except for the one in Model 4, which is statistically insignificant (OR = 1.03, 95% CI: 0.94–1.12; *P* > 0.05).

**Table 2:** Logistic regression for hospital beds and ED admissions due to diabetes-related ACSCs, without clustering.

	**Model 1**	**Model 2**	**Model 3**	**Model 4 (full model)**
	**OR (95% CI)**	**OR (95% CI)**	**OR (95% CI)**	**OR (95% CI)**
**Key independent variable**				
Hosp beds per 1,000 populations	**0.94** ^***^ **(0.94, 0.95)**	**1.01 (0.99, 1.02)**	**1.01 (1.00, 1.02)**	**1.12** ^***^ **(1.09, 1.14)**
**Years**				
Year 2012	0.98^**^ (0.96, 0.99)	0.99 (0.97, 1.01)	0.99 (0.97, 1.01)	0.99 (0.97, 1.00)
Year 2013	0.94^***^ (0.93, 0.96)	0.96^***^ (0.95, 0.98)	0.96^***^ (0.94, 0.98)	0.96^***^ (0.94, 0.97)
Year 2014	0.91^***^ (0.90, 0.93)	0.94^***^ (0.92, 0.95)	0.93^***^ (0.92, 0.95)	0.93^***^ (0.92, 0.95)
Year 2015	0.93^***^ (0.91, 0.95)	0.95^***^ (0.94, 0.97)	0.95^***^ (0.94, 0.97)	0.95^***^ (0.93, 0.96)
**Individual factors**				
Age (ref.: 20–49)	X			
50–64	X	1.20^***^ (1.18, 1.23)	1.21^***^ (1.18, 1.23)	1.21^***^ (1.18, 1.23)
65–79	X	1.34^***^ (1.32, 1.37)	1.34^***^ (1.32, 1.37)	1.34^***^ (1.32, 1.37)
80+	X	1.45^***^ (1.43, 1.48)	1.45^***^ (1.42, 1.48)	1.46^***^ (1.43, 1.48)
Sex (ref: male)	X	0.95^***^ (0.94, 0.96)	0.95^***^ (0.94, 0.96)	0.95^***^ (0.94, 0.96)
Township with high aborigines (ref: < 75th percentile)	X	0.90^***^ (0.89, 0.91)	0.90^***^ (0.89, 0.91)	0.89^***^ (0.87, 0.90)
Low income (ref: non-low income)	X	1.28^***^ (1.24, 1.32)	1.27^***^ (1.23, 1.31)	1.27^***^ (1.23, 1.31)
Percentage of individuals without a high school diploma	X	2.51^***^ (2.35, 2.69)	2.35^***^ (2.19, 2.51)	1.75^***^ (1.61, 1.91)
Charlson comorbidity index	X	0.93^***^ (0.93, 0.93)	0.93^***^ (0.93, 0.93)	0.93^***^ (0.93, 0.93)
**Hospital characteristics**				
Regional hospital (ref: Medical center)	X	X	1.20^***^ (1.19, 1.22)	1.20^***^ (1.18, 1.22)
District hospital (ref: Medical center)	X	X	1.05^***^ (1.04, 1.07)	1.04^***^ (1.03, 1.06)
**Community characteristics**				
Primary care physicians per 1,000 populations	X	X	X	0.43^***^ (0.36, 0.50)
Suburban (ref: urban)	X	X	X	0.99 (0.97, 1.00)
Rural (ref: urban)	X	X	X	1.11^***^ (1.08, 1.14)

^*^P < 0.05.

^**^P < 0.01.

^***^P < 0.001.

**Table 3:** Logistic regression for hospital beds and ED admissions due to diabetes-related ACSCs, with clustering patients at the hospital and community level.

	**Model 1**	**Model 2**	**Model 3**	**Model 4 (full model)**
	**OR (95% CI)**	**OR (95% CI)**	**OR (95% CI)**	**OR (95% CI)**
**Key independent variable**				
Hosp beds per 1,000 populations	**0.95** ^***^ **(0.92, 0.97)**	**0.99 (0.95, 1.03)**	**0.99 (0.95, 1.03)**	**1.03 (0.94, 1.12)**
**Years**				
Year 2012	0.97^*^ (0.95, 0.99)	0.98 (0.96, 1.00)	0.98 (0.96, 1.00)	0.98 (0.96, 1.00)
Year 2013	0.95^***^ (0.92, 0.98)	0.96^*^ (0.93, 0.99)	0.96^*^ (0.93, 0.99)	0.96^**^ (0.93, 0.99)
Year 2014	0.94^***^ (0.90, 0.97)	0.95^**^ (0.91, 0.98)	0.95^**^ (0.91, 0.98)	0.95^**^ (0.91, 0.98)
Year 2015	0.94^**^ (0.91, 0.98)	0.96^*^ (0.92, 0.99)	0.96^*^ (0.92, 0.99)	0.95^*^ (0.91, 0.99)
**Individual factors**				
Age (ref.: 20–49)	X			
50–64	X	1.19^***^ (1.17, 1.22)	1.19^***^ (1.17, 1.22)	1.19^***^ (1.17, 1.22)
65–79	X	1.32^***^ (1.29, 1.35)	1.32^***^ (1.29, 1.35)	1.32^***^ (1.29, 1.35)
80+	X	1.45^***^ (1.41, 1.50)	1.45^***^ (1.41, 1.50)	1.45^***^ (1.41, 1.50)
Sex (ref: male)	X	0.95^***^ (0.94, 0.96)	0.95^***^ (0.94, 0.96)	0.95^***^ (0.94, 0.96)
Township with high aborigines (ref: < 75th percentile)	X	0.99 (0.94, 1.04)	0.99 (0.94, 1.04)	0.98 (0.93, 1.04)
Low income (ref: non-low income)	X	1.17^***^ (1.14, 1.21)	1.17^***^ (1.14, 1.21)	1.17^***^ (1.14, 1.21)
Percentage of individuals without a high school diploma	X	1.68^***^ (1.40, 2.02)	1.69^***^ (1.41, 2.03)	1.46^***^ (1.16, 1.84)
Charlson comorbidity index	X	0.94^***^ (0.94, 0.94)	0.94^***^ (0.94, 0.94)	0.94^***^ (0.94, 0.94)
**Hospital characteristics**				
Regional hospital (ref: Medical center)	X	X	1.17^***^ (1.12, 1.22)	1.17^***^ (1.12, 1.22)
District hospital (ref: Medical center)	X	X	1.13^***^ (1.08, 1.19)	1.13^***^ (1.08, 1.19)
**Community characteristics**				
Primary care physicians per 1,000 populations	X	X	X	0.84 (0.45, 1.57)
Suburban (ref: urban)	X	X	X	1.05 (1.00, 1.12)
Rural (ref: urban)	X	X	X	1.07 (0.99, 1.15)

^*^P < 0.05.

^**^P < 0.01.

^***^P ≤ 0.001.

The odds ratios among covariates in the full models with and without clustering are similar. The odds ratios for the years 2013–2015 were less than one and significant (OR: 0.93–0.96, 95% CI: 0.91–0.99; *P* < 0.001), indicating that the likelihood of ED hospitalizations was lower than that in the year 2011. The odds ratios of the three age dummies (50–64, 65–79, and 80+, with < 50 as the reference group) ranged from 1.21 to 1.45 (95% CI: 1.18–1.50; *P* < 0.001). Female patients were less likely to have ED hospitalizations than male (OR = 0.95, 95% CI: 0.94–0.96; *P* < 0.001). Patients with low income (OR: 1.17–1.27; 95%CI: 1.14–1.31; *P* < 0.001) or without a high school diploma (OR: 1.46–1.75; 95%CI: 1.16–1.91; *P* < 0.001) had a higher likelihood of ED hospitalizations than their counterparts. Regional and district hospitals had a higher likelihood of ED hospitalizations than medical centers (OR: 1.04–1.20, 95% CI: 1.03–1.22; *P* < 0.001). However, the odds ratio of Aboriginal individuals, the number of primary care physicians per 1,000 populations, and rural areas were significant in the model without clustering but became insignificant in the model with clustering. Unexpectedly, the odds ratio of the Charlson comorbidity index showed that increases in the index were negatively associated with the likelihood of ED admissions (OR = 0.93–0.94; 95%CI: 0.93–0.94; *P* < 0.001).

## Discussion

### Summary of the findings

Our study revealed a positive relationship between the supply of hospital beds and the likelihood of ED hospitalizations due to diabetes-related ACSCs; however, the association was statistically significant without a clustering function but was statistically insignificant with a clustering function after controlling for covariates. Among all covariates, the odds ratios in several variables in the models with and without clustering were consistent. Notably, variables with odds ratios higher than one and statistical significance included age groups, low income, a percentage of individuals without a high school diploma, and regional and district hospitals. Conversely, variables with odds ratios of less than one and statistical significance included dummies for study years, sex (female), and the Charlson comorbidity index.

### Comparison with other studies

Although some programs in the United States and several countries gradually adopted a global budget to pay providers (32), evidence primarily focuses on the effect of a global budget on service volume or expenditure. In the United States, Massachusetts State adopted the Alternative Quality Contract (AQC), and Maryland State adopted the Maryland Total Cost of Care Model, a global budget with providers keeping the margin and incentivizing for quality of care. Both models reduced utilizations (e.g., laboratory tests and ED hospitalizations and visits) while improving quality of care (e.g., tobacco cessation and the tests of glycated hemoglobin and cholesterol for chronic disease management) (5, 33–39). The Centers for Medicare and Medicaid Services launched different types of accountable care organizations that are responsible for managing a patient population within a capped budget. Findings showed that accountable care organizations reduced inpatient use and ED visits and improved preventive care and chronic disease management (40). Outside of the United States, physicians in Germany increased their service volume after Germany applied a global budget with price adjustments on ambulatory care (4). In Denmark, hospitals funded via a global budget with value-based incentives were able to reduce the length of stay for inpatients (41).

Literature has shown that the number of hospital beds per 1,000 populations has been reduced in several OECD countries, such as Australia, Canada, Denmark, France, and Sweden, regardless of whether the countries implemented a global budget (41, 42). In the United States, the number of hospital beds per 1,000 populations in Maryland State has been reduced after implementing the Total Cost of Care Model (43). Given the recent rising number of hospital beds per 1,000 populations from 4.3 to 4.5 in Taiwan, the positive findings in our study and the increases in service volume and intensity of care found in previous studies in Taiwan (7, 10, 11, 25) indicate that the likelihood of ED hospitalizations would be increased when hospital beds are available.

In addition to the supply of hospital beds, social determinants, including income and education at the patient level and hospital characteristics at the hospital level, are positively associated with the likelihood of ED hospitalizations. On average, the ED hospitalizations rate in the current study was 59% (597,476/566,472 in Figure 1). Identifying high-risk patients and conducting interdisciplinary collaboration to meet their social and health needs would reduce ED visits and hospitalizations while keeping patients safe at home.

### Limitations

The present study has limitations. First, the study used data from 2011 to 2015. The majority of the expanded hospital beds are still under reconstruction. Our findings cannot completely reflect the association when all new hospital beds are available in the market. Furthermore, the NHIA has launched several interventions, such as increasing the differences in co-payment and encouraging mutual referrals among medical centers, district hospitals, and local hospitals to reduce the burden on medical centers and strengthen local hospitals and clinics in the communities. Utilizing the most recent data to investigate the relationship between hospital beds and ED hospitalizations is encouraged. Second, the AHRQ PQI program excludes patients transferred from other institutions or receiving specific procedures. Our data did not have the codes for procedures and transfer from a hospital or other institutions; therefore, the number of diabetes-related ACSCs was likely be overestimated. However, the limitations of missing these variables in the data are unlikely to affect our findings since we applied the same rules to extract diabetes-related ACSCs from inpatient and outpatient files. Third, we assume that hospitals are engaging in the game of prisoner's dilemma. However, hospitals may adjust their ED hospitalizations policy to maximize their margin based on their historical reimbursement data, which cannot be detected by the current study, although the chance is low given the existing evidence from Taiwan (9–11, 25). Finally, the present study focused on diabetes-related ACSCs. Future studies examining other discretionary conditions (e.g., heart failure or asthma) and non-discretionary conditions (e.g., fracture or heart attack) are also recommended.

### Policy implications

Despite the limitations, our findings have policy implications. First, the fast-expanding number of hospital beds in recent years may motivate hospitals to admit ED patients with preventable and discretionary conditions under a global budget with a floating-value payment system. Therefore, the call to increase the floating-point value to one NTD would eventually challenge the financial sustainability of the NHI without adding value to patient care. Given the evidence from other countries, adding value-based incentive mechanisms to the current global budget system is strongly recommended. Second, although 99% of the population in Taiwan is enrolled in the NHI, patients with low income and without high school education are at risk of ED hospitalizations due to diabetes-related ACSCs. Policies that effectively address the risks of social determinants contributing to poor health outcomes are strongly recommended, which would have the potential to significantly improve population health while reducing the financial burden on the NHIA.

## Conclusion

An increase in the number of hospital beds is likely to motivate providers to admit ED patients with preventable and discretionary conditions under hospital global budgeting with a floating-point value mechanism, which would lead to high hospitalization rates without adding value to patient care. Adding value-based incentive mechanisms to the current global budget payment and addressing social risk factors contributing to poor health outcomes would improve population health and reduce preventable hospitalizations while improving the financial sustainability of the NHI.

## Data Availability

The datasets presented in this article are not readily available because the datasets used and analyzed in the present study are not publicly available but are available from the Taiwan National Health Insurance Program in Taiwan. Restrictions apply to the availability of these data, which were used under license for the present study. Requests to access the datasets should be directed at: https://nhird.nhri.org.tw with the permission of the Taiwan National Health Insurance (NHI) program.
